# Symplastic Hemangioma of the Limbal Conjunctiva in a Pediatric Patient With Xeroderma Pigmentosa: A Case Report and Review of Literature

**DOI:** 10.1002/ccr3.72885

**Published:** 2026-06-10

**Authors:** Mahir Mahmood, Kanwal Babar, Saira Rathore, Samina Zaman, Aalaa Saleh

**Affiliations:** ^1^ Chughtai Institute of Pathology Lahore Pakistan; ^2^ Faculty of Medicine, Lebanese University Beirut Lebanon

**Keywords:** limbal conjunctiva, pediatric ocular lesions, symplastic hemangioma, vascular tumors, xeroderma pigmentosa

## Abstract

Symplastic hemangioma (SH) is a rare benign vascular lesion marked by striking nuclear atypia in non‐endothelial stromal cells, often mimicking malignancy. It is typically seen in older adults, with only four cases reported to date. Pediatric presentations are exceedingly rare. We present a unique case of limbal SH in a 7‐year‐old female with xeroderma pigmentosa (XP), who presented with a pigmented conjunctival mass clinically suspected to be malignant. Given the high risk of malignancy in patients with XP—including squamous cell carcinoma and melanoma—SH can pose a diagnostic dilemma. Histological evaluation revealed a polypoidal lesion covered by stratified squamous epithelium, with numerous small blood vessels lined by plump endothelial cells and interspersed bizarre, atypical stromal cells. No necrosis, atypical mitosis, or definitive features of malignancy were identified. Immunohistochemistry showed SMA positivity in the atypical cells, confirming vascular origin, while the Ki‐67 index was very low (< 1%), supporting a benign nature. These findings ruled out malignancy despite alarming histological features. To our knowledge, this is the first reported case of limbal SH in a pediatric patient with XP. This case highlights the importance of histopathological and immunohistochemical analysis in distinguishing SH from aggressive vascular tumors and avoiding unnecessary aggressive treatment. It adds valuable insight into the rare spectrum of vascular lesions in XP.

## Key Clinical Message

Symplastic hemangioma is a rare benign vascular lesion that can mimic malignancy, especially in patients with xeroderma pigmentosum. Recognition of its atypia without endothelial dysplasia prevents misdiagnosis and avoids unnecessary aggressive treatment, even in pediatric limbal presentations where clinical suspicion is naturally heightened.

## 1 Introduction

Symplastic hemangioma (SH) is a rare benign vascular lesion characterized histologically by prominent nuclear atypia in non‐endothelial components such as stromal or smooth muscle cells, often mimicking malignancy [1]. Typically reported in older individuals, only four cases have been documented in the literature to date and pediatric presentations are exceedingly rare [1, 2, 3, 4]. SH shows degenerative pleomorphism, with atypical changes in the vascular smooth muscle and stromal cells of an existing vascular lesion [4, 5]. In surgical pathology, the term “symplastic” has been used for lesions that display striking, often multinucleated cellular forms without meeting the usual microscopic criteria for malignancy [6]. Although SH is a benign superficial proliferation of blood vessels, its appearance can resemble that of a malignant process. The key feature that separates it from true malignant vascular tumors is the absence of endothelial nuclear atypia. SH often poses significant diagnostic challenges, particularly in settings of XP where the risk of malignant neoplasms like squamous cell carcinoma, basal cell carcinoma, and melanoma is high. We report a unique case of limbal SH in a 7‐year‐old female with xeroderma pigmentosa (XP), presenting as a pigmented conjunctival mass with clinical suspicion of malignancy.

## 2 Case Presentation

A 7‐year‐old female who is a known case of XP presented with a gradually enlarging, non‐painful, pigmented mass over the limbal conjunctiva of the left eye for the last 2 months. The lesion had been slowly increasing in size, without any associated bleeding or vision disturbances, and there is no history of weight loss. There was no prior history of ocular malignancy. Clinical examination in the setting of XP raised suspicion of malignancy for which an excision was planned. On ophthalmic examination, visual acuity was within normal limits for age. Extraocular movements were full and unrestricted in all gazes. Intraocular pressure was within normal physiological limits. The gross specimen consists of tiny blackish brown polypoidal tissue fragments measuring 0.8 × 0.6 × 0.5 cm.

**FIGURE 1:**
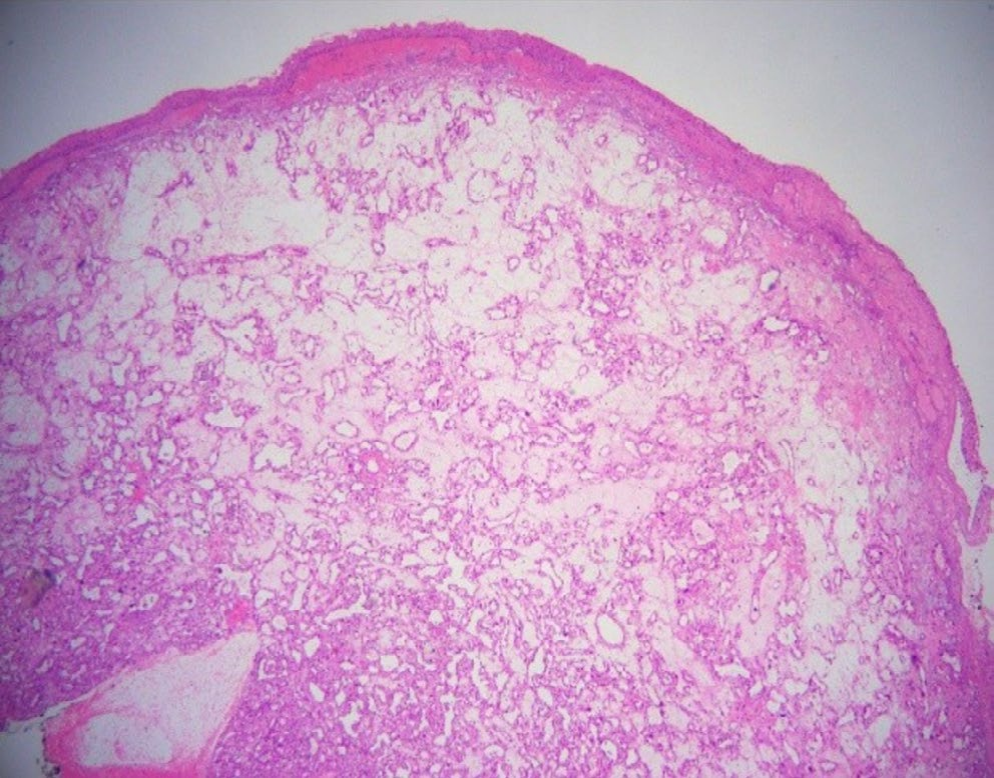
Low‐power view (H&E stain) of the conjunctival lesion showing prominent dilated vascular spaces.

**FIGURE 2:**
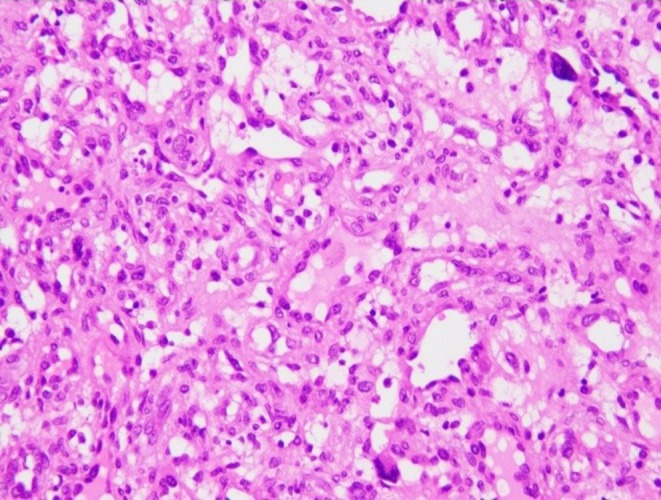
High magnification reveals dilated vascular channels with hyperchromatic bizarre nuclei atypia.

**FIGURE 3:**
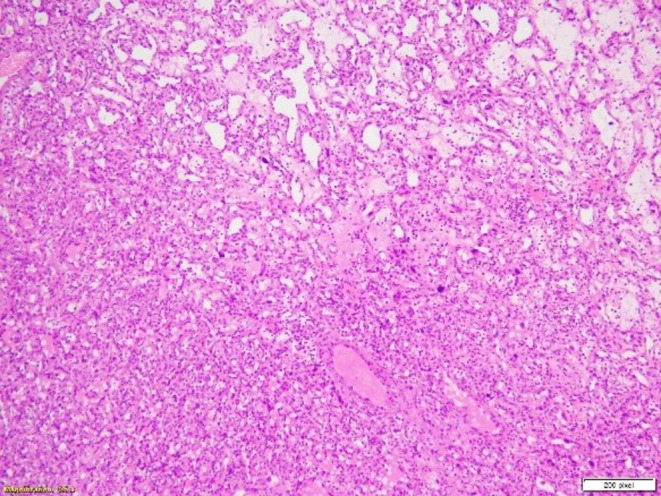
Low‐power view (H&E stain) of the conjunctival lesion showing atypical stromal cells and ectatic blood vessels within a fibrous stroma.

Histopathological evaluation revealed a polypoidal mass arranged in lobular configuration covered by stratified squamous epithelium and composed of a vascular lesion. Numerous small blood vessels lined by plump but hyperchromatic endothelial cells. Notably, interspersed bizarre‐shaped atypical stromal cells were observed. No mitotic figures, necrosis, or features of malignancy were seen. These changes raised a differential of a vascular lesion with cellular atypia (Figures 1, 2, 3).

**FIGURE 4:**
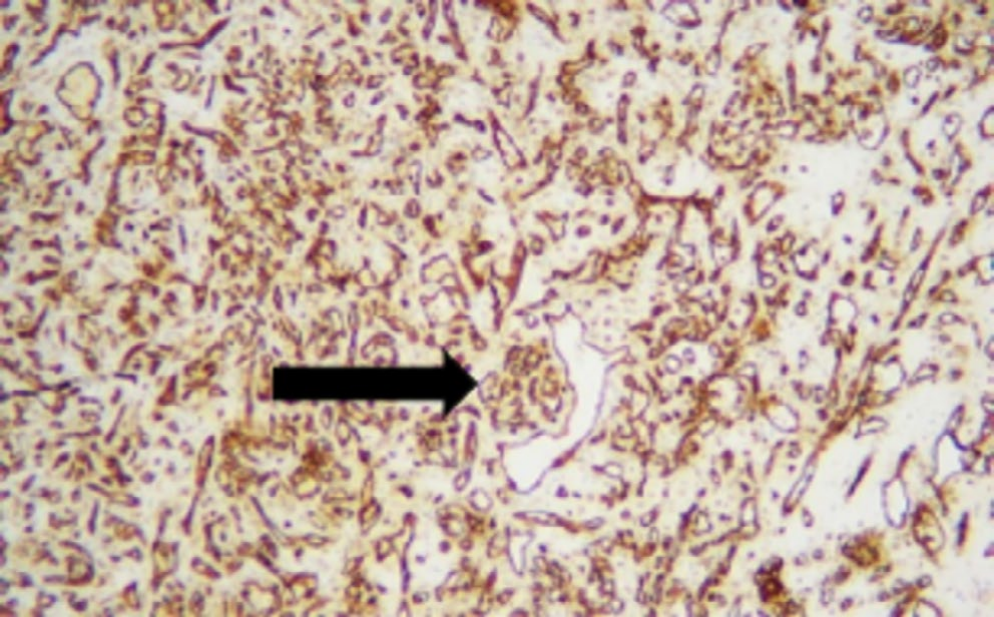
Immunohistochemical staining highlights the endothelial lining of the vascular spaces and bizarre nuclei confirming the vascular nature of the lesion.

Immunohistochemical staining showed positivity for smooth muscle actin (SMA). The lesion was negative for CD31, CD34, and desmin. The Ki‐67 proliferative index was extremely low (less than 1%, indicating very low mitotic activity). Cytomegalovirus (CMV) immune‐stain was negative. The overall histopathological and immunohistochemical features were consistent with symplastic hemangioma (Figure 4).

## 3 Discussion

Symplastic hemangioma (SH) is a rare and benign vascular lesion distinguished by its deceptive histological features. It is characterized by degenerative nuclear atypia in non‐endothelial cells, typically within vascular smooth muscle or stromal cells. Endothelial cells remain cytologically bland, a feature that helps differentiate SH from true vascular‐like angiosarcoma [1, 4].

To date, only four cases of SH have been reported in the medical literature, all occurring in adult patients, mostly females. These include cutaneous (2 cases), calvarial and intracranial presentations as shown in Table 1. Out of the four cases, three were seen in female patients and one was in male. This case represents the first known case of SH arising in the limbal conjunctiva of a pediatric patient, who is the known case of XP—a rare autosomal recessive condition associated with defective DNA repair mechanisms and increased cancer susceptibility.

**TABLE 1:** Previous case reports on symplastic hemangiomas.

Sr. no	References	Age	Sex	Site
1	Current case	7 year	Female	Left eye conjunctiva (size: 8 mm)
2	Goh SG et al. (2006)	47 Year	Female	Left scapula
3	Bantaan NAA, et al. (2021)	Adult (age not specified)	Female	Multiple calvarial sites (left parietal and occipital, right parietal)
4	Ando K, Ogiwara H, et al. (2019)	Adult (age not specified)	Male	Intracranial (side not specified)
5	Jain D, Sharma Mc, et al. (2007)	Adult (age not specified)	Female	Scalp (side not specified)

Given the rarity of SH and its deceptive appearance, size can be a useful parameter for clinicians and pathologists to consider when evaluating histology to prevent misdiagnosis and overtreatment, including unnecessary radical surgery or adjunctive therapies. The lesion in this case is 0.8 × 0.6 × 0.5 cm while other reported cases' size ranges from 0.6 to 0.8 cm. Accurate diagnosis hinges on careful morphological assessment supplemented with targeted immunohistochemistry.

The histopathological challenge in SH lies in the presence of bizarre, atypical nuclei, which can resemble high‐grade sarcomas or malignant vascular tumors specially in older age groups. In patients with XP, the threshold for malignancy is understandably lower, making accurate diagnosis critical [1]. In our case, although the stromal cells exhibited marked atypia, their small size, lobulated architecture, absence of mitotic activity, and low Ki‐67 index supported the benign nature of the lesion. SMA positivity in the bizarre‐shaped cells further confirmed their endothelial origin, aiding in the diagnosis of SH. The presence of atypical symplastic endothelial cells favors symplastic hemangioma over simple congenital hemangioma. The absence of atypical mitotic figures and slit‐like vascular spaces, along with the patient's age, makes Kaposi sarcoma an unlikely diagnosis.

This case contributes valuable new information to the sparse literature on SH and expands the known age range of affected individuals. Its occurrence in a pediatric patient with XP is unprecedented and serves as a reminder to consider SH in the differential diagnosis of limbal conjunctival masses, especially in patients predisposed to malignancies [2, 3].

## 4 Conclusion

Symplastic hemangioma, though rare and histologically alarming, remains a benign vascular lesion. Its marked nuclear atypia can closely mimic malignant vascular tumors, creating a significant diagnostic challenge. Careful evaluation of histopathological features supported by immunohistochemical analysis is essential to distinguish benign degenerative atypia from true malignancy. This distinction is particularly important in high‐risk settings such as xeroderma pigmentosum, where unusual cutaneous lesions may raise concern for aggressive neoplasms. Awareness of symplastic hemangioma in pediatric patients with XP is therefore critical to prevent overdiagnosis and to ensure appropriate clinical management.

## Author Contributions

**Mahir Mahmood:** conceptualization, software, visualization, writing – original draft, writing – review and editing. **Kanwal Babar:** writing – original draft, writing – review and editing. **Saira Rathore:** writing – original draft, writing – review and editing. **Samina Zaman:** writing – original draft, writing – review and editing. **Aalaa Saleh:** writing – review and editing.

## Funding

The authors have nothing to report.

## Ethics Statement

Ethics approval was not required because it was a case report.

## Consent

Written informed consent was taken from the patient for this case report prior to publishing it. Final manuscript was reviewed by all the authors, and consent was taken before publishing it.

## Conflicts of Interest

The authors declare no conflicts of interest.

## Data Availability

Data available on request from the authors.
